# Selfish

**Published:** 1867-03

**Authors:** 


### SELFISH.

The writer of this is under so many obligations to his professional friends, and others, for the many favors and evidences of sympathy received during and since his recent severe illness, that he finds both tongue and pen utterly inadequate to a proper expression of his thoughts and feelings. May the blessings of Him who created friendship, be showered down on the heads of those who so divinely practice it! W.

